# The Jak/Stat Signaling Pathway Is Downregulated at Febrile Temperatures

**DOI:** 10.1371/journal.pone.0049374

**Published:** 2012-11-14

**Authors:** Tobias Nespital, Ger J. Strous

**Affiliations:** Department of Cell Biology and Institute of Biomembranes, University Medical Center Utrecht, Utrecht, The Netherlands; Universidade Federal do Rio de Janeiro, Brazil

## Abstract

**Background:**

The Janus family of kinases (JAKs), Jak1, Jak2, Jak3, and Tyk2, constitute a subgroup of non-receptor protein tyrosine kinases. Upon cytokine binding, the receptor-associated kinases are activated and phosphorylate tyrosine residues in their cognate cytokine receptors. Their activities are controlled at several levels and include cellular concentration, auto-activation, and degradation.

**Principal Findings:**

Our findings show that elevated temperatures in the fever range irreversibly aggregate Jak2 and considerably reduce functional Jak2 protein levels. Jak2 synthesis remains unaltered. We observed that also the protein level of the signal transducer and activator of transcription, STAT5b, is transiently decreased at temperatures above 37°C. Consequently, the signaling response, e.g. via the growth hormone receptor, is reduced.

**Conclusions/Significance:**

These findings predict that elevated body temperatures lower the responsiveness of cytokine receptors.

## Introduction

Fever is a common response of the body to infection and injury, tightly regulated by the balance between endogenous mediators known as cytokines that act either as pro-inflammatory/pyrogenic (interleukin (IL)-1β; IL-6, tumor necrosis factor α (TNFα)) or anti-inflammatory/cryogenic intermediaries (e.g. IL-10 and the IL-1 receptor antagonist (IL-1ra) [1]–[6]. The JAK family plays a critical role in growth, development, survival and differentiation, especially of immune and hematopoietic cells through signal transduction of many cytokine receptors [7]. Upon cytokine binding, changes in the structure of the receptors initiate (trans)phosphorylation and JAK/STAT signal transduction. Each cytokine receptor is regulated by specific JAK-STAT combinations, whereby the sensitivity to cytokine stimulation and the gene expression are cell-type related [8]. Besides the JAK/STAT pathway, JAKs can initiate other pathways like the mitogen-activated protein kinases pathway and the phosphoinositol 3-kinase pathway [9]. The JAK family members consist of seven highly conserved JAK homology domains (JH1-7), including a kinase (JH1) and an N-terminal FERM domain, which binds to the box-1 sequence in cytokine receptors [10]. The JAK/STAT signaling pathway is regulated through various mechanisms [11], [12].

Jak2 binding to cytokine receptors such as prolactin (PRLR), erythropoietin (EpoR), thrombopoietin, growth hormone (GH receptor), and the IL-5 receptor stabilizes them at the cell surface [13]–[17]. Thus, in the absence of ligand, Jak2 keeps the receptors at the cell surface, maintaining cytokine sensitivity, while, in the presence of cytokine, it starts the signal transduction and induces rapid receptor degradation. Therefore, the homeostasis of Jak2 serves an important role in the cytokine sensitivity of cells. Although JAK family members are stable proteins, the regulation of their homeostasis may depend on external stressors.

Thermal stress occurs in mammals as a regulated defensive response of fever upon pathogenic stimulation, whereas hyperthermia is unregulated and considered as only one aspect of fever [18]. Under fever conditions, different kinds of endogenous anti-inflammatory cytokines are induced, both pyrogenic such as IL-1α and -β, IL-6, IL-8 and interferon-γ (IFNγ), and antipyretic, such as IL-10 and TNFα. A multitude of interactions between pyrogenic and antipyretic cytokines as well as a variety of other factors is involved in the fever response. However, little is understood about the underlying molecular mechanisms [19], [20]. To demonstrate the universal character, we used a variety of cell lines as well as peripheral blood mononuclear cells (PBMC) to show that the JAK/STAT signaling route contains thermo-labile factors. In particular, the levels of Jak2 are decreased at febrile temperatures. Moreover, we found that thermal stress lowers the protein levels of other JAK family members as well as of STAT5b. We conclude that the Jak2/STAT5 signaling pathway is downregulated at fibril temperatures. Furthermore, we found clear indications that at 40°C Jak2 rapidly and irreversibly aggregates in a kinase activity-dependent manner.

## Results

### Under thermal stress, Jak2 is degraded in an ubiquitin-dependent manner

**Figure 1:**
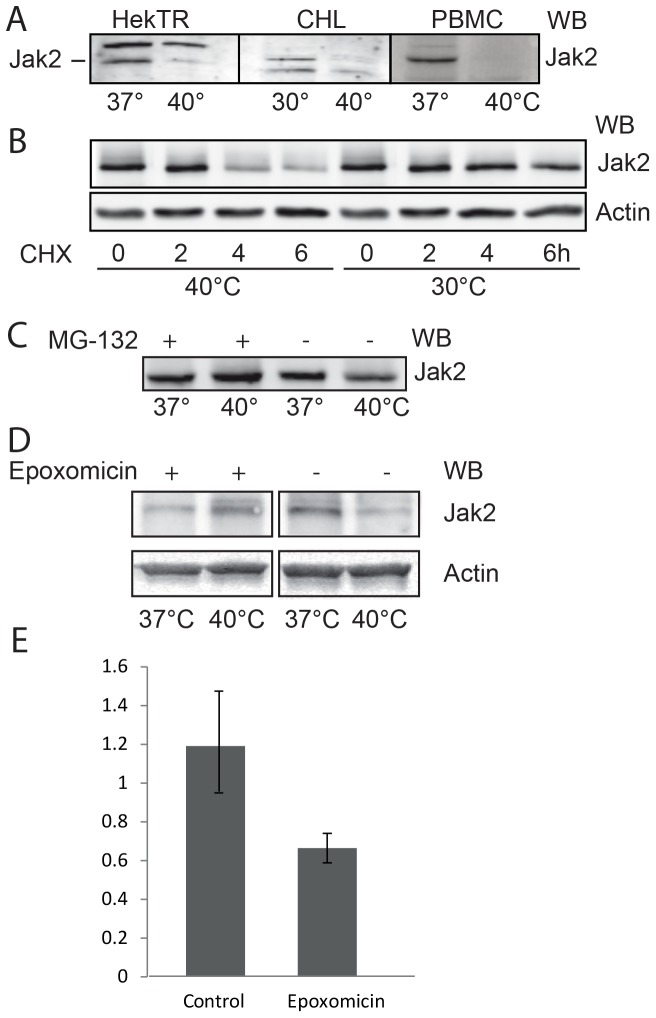
Effect of stressors on Jak2 protein levels in various cell types. (**A**) Hek293-TR, Chinese hamster lung and PBM cells were incubated for 3 h, as indicated. The cells were lysed in 1% Triton X-100 and equal aliquots of the lysates were analyzed by western blotting (WB) for endogenous Jak2, using anti-Jak2 antibody. The antibody recognized a-specific bands in lysates of HekTR (above Jak2) and CHL (below Jak2). (**B**) In the presence of 20 µg/ml cycloheximide (CHX) Chinese hamster cells were transiently transfected with Jak2 and incubated at either 37 or 40°C. The cells were lysed in 1% SDS and equal aliquots were analyzed (WB) using anti-Jak2 and anti-actin antibodies. (**C**) Equal amounts of isolated PBMCs were incubated with or without 20 µM MG-132 at 37 or 40°C for 4 h. The cells were lysed in 1% SDS and equal amounts were analyzed (WB) using anti-Jak2 antibody. (**D**) Effect of Epoxomicin on Jak2 protein levels in PBMCs. Equal amounts of isolated PBMCs were incubated with or without10 µM Epoxomicin at 37 or 40°C for 4 h. The cells were lysed in 1% SDS and equal amounts were analyzed (WB) using anti-Jak2 antibody. The data in (A, B, C, D) are representative of three independent experiments. (**E**) Quantification of (D). The ratio of Jak2 to actin signals (western blot) were calculated. The data represent the mean of three independent experiments ± SEM.

In this study, we investigated the influence of thermal stress in JAK homeostasis and activity. As seen in Fig. 1A, the level of endogenous Jak2 in non-ionic detergent-containing lysates from Hek293-TR (human), Chinese hamster lung cells as well as from human PBMCs were strongly decreased at 40°C compared to 37 or 30°C. Steady state Jak2 protein levels were the same at 37 and 30°C. To show that protein synthesis was not inhibited, the Chinese hamster lung cells were transfected with Jak2 and treated with the protein biosynthesis inhibitor cycloheximide (CHX) at 30 and 40°C for 2–6 h (Fig. 1B). No decrease of Jak2 levels was observed at 30°C within this period, indicating that Jak2 is a stable protein, whereas at 40°C, under cycloheximide treatment, the levels of Jak2 decreased significantly. Next, we investigated, whether the disappearance of Jak2 at 40°C was due to increased proteasomal degradation by treating PBMCs with the proteasome inhibitors, MG-132 and Epoxomicin. To ascertain that we monitored the total protein content the cells were lysed in SDS. As seen in Fig. 1C–E, a 4 h-treatment at 40°C caused a clear and reproducible decrease in Jak2 protein levels, while no change occurred in presence of either MG-132 or Epoxomicin. We conclude that the disappearance of Jak2 at elevated temperature is due to (proteasomal) degradation.

### Cytokine sensitivity is reduced

**Figure 2:**
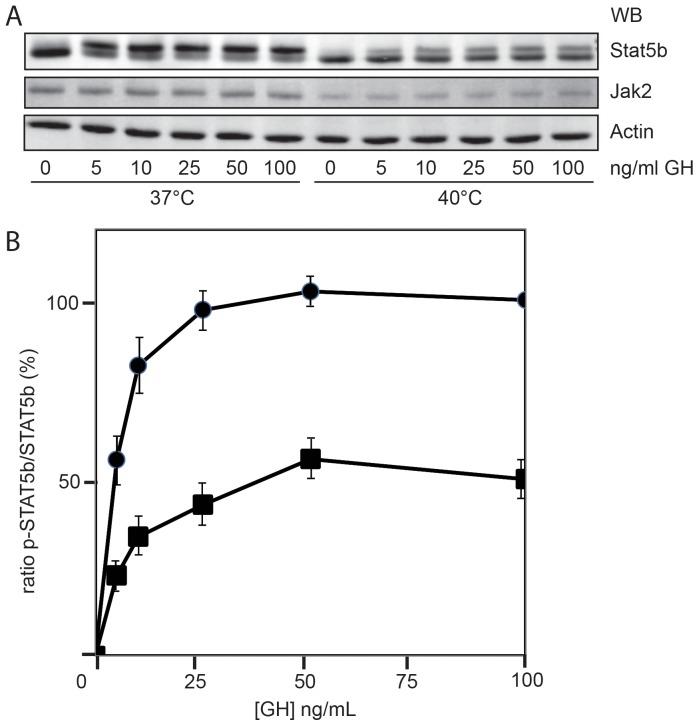
JAK/STAT signaling response is diminished under elevated temperature. (**A**) Mouse 3T3-F442A preadipocytes were incubated at 37 or 40°C for 4 h and then treated with the indicated concentrations of GH for 10 min. The cells were lysed in 1% SDS and equal amounts of lysate were analyzed by western blotting (WB), using anti-Jak2, anti STAT5b and anti-actin antibodies. (**B**) Quantification of Fig. 2A. At each point the ratio of the lower band to the total STAT5b signal was calculated (100 ng/ml GH at 37°C was set 100%; 0 ng/ml was set 0%). The data represent the mean of four independent experiments 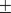 SEM. (*circles*, 37°C; *squares*, 40°C).

Once established that increased temperatures cause decreased Jak2 protein levels, we asked whether thermal stress also reduces signaling via the JAK/STAT pathway. We utilized the endogenous GH receptor system of mouse 3T3-F442A preadipocytes. These cells contain high numbers of endogenous GH-receptors and are therefore suitable for studying (GH-induced) Jak/STAT signaling. Cells were incubated at 37 and 40°C for 4 h and increasing concentrations of GH were added for 10 min. STAT5b became phosphorylated, which resulted in a 2-kDa increase in apparent molecular weight. Whereas at 37°C this shift reached its maximum at 5 ng/ml GH, at 40°C STAT5b never reached maximal stimulation (Fig. 2A, quantified in panel B). Fig. 2A also shows that, in addition to the cellular levels of Jak2, STAT5b was decreased as well. Also in PBMCs and Hek293-TR cells endogenous STAT5b levels were reduced at 40°C. Thus, incubation at 40°C lowered the signaling capacity of cells considerably, probably due to the reduction of both Jak2 and STAT5b levels.

### Under thermal stress, Jak2 forms irreversible aggregates

**Figure 3:**
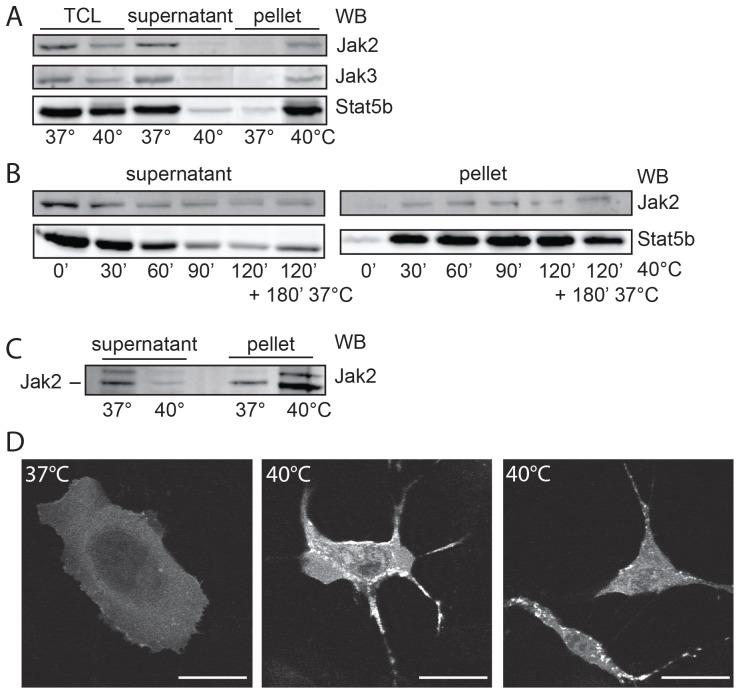
Thermal stress induces both Jak2 degradation and aggregation. (**A**) Equal amounts of isolated PBMCs were incubated at 37°C and 40°C, respectively, for 4 h. The cells were either lysed in 1% SDS and analyzed in western blot (TCL) or lysed in 1% NP-40; the lysates were centrifuged (500 g), the supernatants were collected and the pellets were dissolved in 1% SDS. Equal aliquots were analyzed by western blotting (WB) using anti-Jak2, anti-Jak3 and anti-STAT5b antibodies. (**B**) Equal amounts of isolated PBMCs were incubated at 40°C for the indicated times, lysed in 1% NP-40, and analyzed as in Fig. 3A (WB) using anti-Jak2 and anti-STAT5b antibodies. For the right lanes in the four panels, the incubation was continued for 3 h at 37°C. (**C**) Equal amounts of Hek293-TR cells were lysed in 1% Triton X-100, the lysates were clarified by centrifugation, and incubated at 37°C and 40°C, respectively, for 4 h. Next, the lysates were centrifuged at 500 g, and equal aliquots of supernatant and pellet were analyzed by western blotting (WB), using anti-Jak2 antibody. The upper band is an unspecific background band. (**D**) γ2A Jak2 −/− cells, transfected with GFP-Jak2, were incubated at 37 and 40°C for 4 h and fixed in formaldehyde. Representative pictures are shown. Fluorescence was visualized with a confocal microscope. Bar, 20 µm. All data in this figure are representative of three independent experiments.

Since Triton-soluble Jak2 protein levels were clearly decreased (Fig. 1A), while protein degradation, measured in SDS lysates, occurred only to a limited extend (Fig. 1C), we analyzed the nature of this discrepancy. Fig. 3A confirms that in SDS lysates of PBMCs both Jak2 and STAT5b levels were decreased at 40°C (TCL), most likely due to degradation. We then lysed PBMCs in non-ionic detergent, and analyzed the soluble and insoluble fractions. At 40°C, both Jak2 and STAT5b levels were decreased in the supernatant and enriched in the pellet (Fig. 3A). This transition occurred within 60 min (Fig. 3B). To determine whether this process was reversible we restored the temperature to 37°C for a period of 3 h. As seen in Fig. 3B, right lanes, while Jak2 remained insoluble, STAT5 partly re-solubilized. Together, these results indicate that treatment at 40°C causes rapid insolubility of both Jak2 and Stat5b. In addition to Jak2, also Jak3 became insoluble at 40°C (Fig. 3A). Whether also Jak1 and Tyk2 behave the same, remains an open question, as the endogenous levels of both proteins were below detection level in PBMCs. As the four JAKs are both structurally and functionally similar, most likely, the effect of thermal stress is comparable. To confirm that Jak2 has an intrinsic propensity to become insoluble we lysed PBMCs in a non-ionic detergent and incubated the supernatant for 2 h at 40°C. Again, Jak2 became insoluble (Fig. 3C).

Protein aggregation is an organized process in which aggregates, called aggresomes, are deposited in specific cellular sites. These cytoplasmic inclusion bodies form because of malfolding e.g. due to a point mutation as in cystic fibrosis transmembrane conductance regulator (22). To visualize the aggregation process we analyzed GFP-Jak2-transfected Jak2-deficient human sarcoma γ2A (Jak2−/−) cells with a fluorescence microscope (Fig. 3D). Whereas Jak2 showed an equal distribution in the cytosol at 37°C, at 40°C it occurred in clusters, characteristic of aggresomes. Together with the findings of the fractionation experiments, these results demonstrate that Jak2 aggregates upon temperature stress.

### Kinase activity determines Jak2-aggregation upon thermal stress

**Figure 4:**
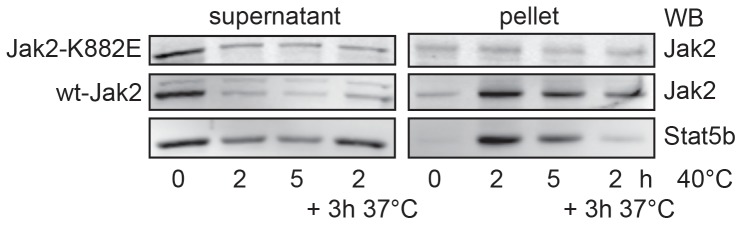
Kinase inactive Jak2 is degraded but not aggregated. γ2A Jak2 −/− were transiently transfected with Jak2-K882E or wild type Jak2, respectively. Equal amounts of cells were incubated at 37 and 40°C, respectively, for the indicated times. The cells were lysed, fractionated and analyzed as in Fig. 3B. The data in this figure are representative of three independent experiments.

To investigate the mechanism of Jak2 inactivation we asked whether Jak2 kinase activity is a prerequisite for aggregation. Cell lysates from γ2A Jak2 −/− cells, transfected with either Jak2-K882E or wild type Jak2 were analyzed (Fig. 4, upper panel). Interestingly, while cytosolic degradation was preserved and wild-type Jak2 became insoluble, no aggregation occurred for kinase-dead Jak2. Apparently, the conformational change caused by the K882E mutation rendered the Jak2 molecule resistant to aggregation. One can speculate on the causality of this finding. Many misfolded proteins expose hydrophobic surfaces that under unstressed conditions are buried in their interior or in the interface with other subunits [21]. Such exposure may lead to alternate, non-native conformations that interact with each other to form aggregates [22]. The K882E mutation in the kinase domain may prevent Jak2 from exposing hydrophobic amino acids. Marubayashi et al. reported a HSP90 inhibitor that can disrupt Jak2 protein stability in polycythemia *vera*, suggesting that the Jak2 V617F mutation depends on HSP90 for its constitutive activity [23]. Apparently, the K882E mutation increases the thermal stability of Jak2. Whether this depends on HSP90 remains to be investigated. In order to establish whether Jak2 and STAT5b can stabilize each other we also co-expressed the two proteins and analyzed their behavior at elevated temperature. Fig. 4, lower panel, shows that this is not the case. Although the two proteins are probably not expressed relative to the situation of the endogenous proteins, this result suggests that aggregation of the two proteins is independent.

## Discussion

In our study, we show that Jak2 protein levels rapidly decrease in cells exposed to thermal stress, while its synthesis remains normal. The analogy of these findings in a variety of cell lines, as well as in PBMCs isolated from human blood, indicate the universal validity of this effect. Although Jak2 is a stable protein, it is degraded in a ubiquitin-dependent manner via the ubiquitin-proteasome pathway [24]. At 40°C also STAT5 protein levels were reduced. The significance of this process was illustrated in mouse 3T3 cells that showed a decreased GH response at 40°C. Jak2 underwent aggregation in an irreversible manner, while STAT5 recovered its solubility upon temperature readjustment. Interestingly, kinase-inactive Jak2 did not show aggregation, although the effect of degradation in the cytoplasm at elevated temperatures was conserved.

The formation of aggresomes is mediated by the adapter histone deacetylase 6 (HDAC6), which binds ubiquitinated proteins to the microtubule motor protein dynein. Thereby, the polyubiquitinated cargo is transported in a dynein-based manner along the microtubule cytoskeleton to the final perinuclear site at the micro tubule-organizing center (MTOC) [25]. Recently, treatment of cells with the Jak2 inhibitor WP1130 has been shown to results in Jak2 K63-polyubiquitination and in accumulation of Jak2 in aggresomes [26], which is consistent with previous studies, demonstrating the occurrence of K63-polyubiquitinated proteins in the detergent-insoluble fraction and in aggresomes [27], [28]. Therefore, it can be speculated that elevated temperatures cause enhanced K63-polyubiquitination, either through increased ubiquitination or decreased deubiquitination, inducing Jak2 aggregation in aggresomes.

Our results are in line with the findings of Rhoads et al. who observed a diminished signaling response to GH in dairy cows, which were exposed to heat stress [29]. Heat stress decreased the levels of GH receptor and GH-dependent STAT5 phosphorylation, and consequently, reduced the GH signaling through STAT5, resulting in decreased hepatic IGF-I mRNA abundance and lower milk production. This complies with our previous observation that Jak2 is a regulator of the steady state levels of GH receptor. As Jak2 inhibits degradation of the receptor, a decrease of cellular Jak2 induces lower GH receptor levels [13]. Together with our current findings, it might explain why fever-range temperatures lower the GH receptor activity and, consequently, slow downstream GH-sensitive bio-energetic processes such as gluconeogenesis.

Environmental factors such as thermal stress can interfere with the folding of proteins in cells [30]. The heat shock proteins HSP70 and HSP90 exhibit ATP-dependent refolding activities [31] that suppress aggregation either by facilitating (re)folding or by inducing their degradation [32]. Jak2 was shown to be a client of the HSP90 [23]. Exhaustion of this quality-control system may result in protein aggregation [33] and subsequent degradation by the proteasome. Furthermore, HSP90 has been implicated in preserving the activity of STAT3 during fever [34]. Although incubation at 39.5°C for 16 h resulted in lower phosphorylation levels, increased activity of HSP90 rescued the STAT3 signaling capacity. Assuming that STAT3 and STAT5b behave similar in febrile conditions, this mechanism might explain our observation that the temperature effect on STAT5b is reversible.

Fever-range thermal stress has complex effects on cytokine activity and synthesis [2]. Together with inflammatory stimuli, like bacterial LPS, febrile temperatures can increase the synthesis of pyrogenic cytokines like IL-6, TNF and IFN-α [35]–[38]. On the other side, thermal stress plays a role in inflammatory responses through the downregulation of cytokine production such as TNF-α and IL-1β [19], [39]. Consequently, reduction of functional Jak2, and consequently downregulated cytokine signaling, might act in the fever response to assure a balanced information response and thereby provides a mechanism of protection against an overload of cytokine signaling. In other instances, JAKs are required for receptor downregulation via endocytosis, as is the case for the IL-5R, in which less Jak2 might up regulate IL-5R signaling capacity [40]. Because of the complexity of the intracellular signaling networks and paradoxical scientific results, further research will determine the specific role of Jak2 in the fever response.

Since JAKs drive many cytokine signaling pathways, their reduced protein levels must have a considerable impact on the signaling events. Under febrile temperatures, some cytokines, such as the pyrogens IL-1, IL-6 and TNF are up regulated. Others with antipyretic effects, such as the anti-inflammatory cytokine IL-10, function as endogenous fever regulators by inhibiting the production of endogenous IL-6 [35]. Thus, a rise in temperature, as it occurs in fever, involves the action of two types of endogenous cytokines, some with pyrogenic and others with antipyretic function. Jak2 levels might act as a second feedback loop. Although we only tested Jak2 activity downstream of the GH receptor, it is very likely that febrile conditions affect the activities of other cytokines that act via Jak2 similarly. The fact that endogenous Jak2 in a variety of tissue culture cells as well as in PBMCs responds similar to febrile temperatures illustrates a universal feature.

Our finding adds a novel element to the already impressive span of control of the JAK family. In addition to being controlled by ancillary factors such as SH2-B [41], their activities are subjected to auto-activation [42]. They stoichiometrically control numbers of certain cytokine receptors at the cell surface [17], [43], they trans-phosphorylate tyrosine residues in specific patterns [44], they detach from activated receptors to be recycled by phosphatases [13], and, as reported here, they respond to heat stress by irreversible aggregation.

## Materials and Methods

### Materials, antibodies and DNA constructs

Monoclonal antibody of Jak2 (AHO1352) was purchased from Invitrogen (Camarillo, CA, USA), monoclonal 4G10 anti-pY from Millipore (Billerica, Ma, USA), anti-actin (Clone C4) from MP Biomedicals Inc. (Amsterdam, The Netherlands), polyclonal anti-STAT5(C-17) (sc-835) from Santa Cruz Biotechnology, Inc. (Santa Cruz, CA, USA) and anti-Jak3 from Millipore (Temecula, CA, USA). The secondary antibodies, goat anti-mouse and goat anti-rabbit IgG Alexa 680, were from Molecular Probes (Eugene, OR, USA) and goat, anti-rabbit and goat anti-mouse IgG IRDye800 were from Rockland Immunochemicals Inc (Gilbertsville, PA). MG-132 (carbobenzoxy-L-leucyl-L-leucyl-L-leucinal) was purchased from Calbiochem-Novabiochem (San Diego, CA, USA) and Epoxomicin from Cayman Chemicals (Ann Arbor, MI, USA). Ficoll-Paque PLUS was bought from GE-Healthcare. Cycloheximide was from Sigma (C7698). Human GH was a gift from Eli Lilly (Indianapolis, IN, USA). Flag-tagged wild-type mouse and GFP-tagged Jak2 constructs were generous gifts from Prof. Carter-Su (University of Michigan, Ann Arbor). The Jak2-K882E mutation was inserted with Quick Change mutagenesis kit from Stratagene (Santa Clara, CA, USA). To construct Jak2-K882E we used forward and reverse primers: 5′-GGGGAGGTGGTCGCTGTAGAAAAGCTTCAGCATAG-3′ and 5′- CTATGCTGAAGCTTTTCTACAGCGACCACCTCCCC-3′.

### Cell culture and transfections

Culture media, fetal calf serum (FCS) and100 units/ml penicillin, 0.1 mg/ml streptomycin (Pen/Strep) were purchased from Gibco (Invitrogen, Groningen, The Netherlands). Human embryonic kidney 293 cells, stably expressing the tetracycline repressor (HEK293-TR), were a gift from Dr. Madelon Maurice (UMC, Utrecht, the Netherlands). The cells were grown in Dulbecco's modified Eagle's medium (DMEM) high glucose (4.5 g/l) and 10% FCS, Pen/Strep and 12 µg/ml Blasticidin S (MP Biomedicals). Chinese hamster lung-ts20 and E36 cells were originally obtained from Dr. Kulka [45]. The cells were transfected with a pCB6 expressing GH receptor construct and cultured in MEMα, supplemented with 10% FCS, 4.5 g/l glucose, Pen/Strep, and 0.45 mg/ml geneticin [46]. Mouse 3T3-F442A preadipocytes were obtained from Dr. Howard Green (Harvard Medical School) and grown in DMEM, supplemented with 5% FCS and Pen/Strep [47]. Human sarcoma γ2A Jak2^−^/− cells were generously supplied by Dr. D.J. Waxman (Boston University, Boston, MA) and maintained in DMEM, supplemented with 1.0 g/L glucose, 10% FCS, and Pen/Strep [13], [48]. DNA transfections were done using FuGene 6 (Roche, Applied Sciences, Almere, the Netherlands). Seventy percent confluent cultures were transfected with 1 µg of DNA in 6-well plates and with 6 µg DNA in 10 cm plates, according to manufacturer's protocol. 24 hours after transfection, cells were used for experiments. Western blotting was carried out as described previously [13]. Where indicated the de novo cell lines, HEK293-TR, Chinese hamster, and γ2A cells, were stably transfected with GHR DNA constructs and were selected according to the rules on genetic modified organisms of the institutional review board of the UMC Utrecht, GGO Project nr: GGO 02-072.

### Isolation of PBMCs

Citrated blood was diluted 1 to 1 (vol/vol) with PBS. 25 ml of blood was added to 15 ml Ficoll-Paque into a 50 ml Falcon tube. The tube was centrifuged at 800 g for 20 min at 18°C. The interface containing the mononuclear cells were harvested and transferred into a 50 ml tube (filled up to 50 ml with PBS). Then the tube was centrifuged at 180 g for 5 min at room temperature, the supernatant was discarded, the pellet was resuspended in 50 ml PBS before centrifuged again at 180 g for 5 min. The cells then were diluted in GIBCO® RPMI Media 1640 (Invitrogen).

### Confocal microscopy

Transfected cells, grown on coverslips, were washed with PBS and fixed for 30 min in 4% paraformaldehyde in PBS. After fixation, the cells were embedded in Mowiol. Representative confocal pictures were taken using LSM510meta from Carl Zeiss, with 63× n/a 1.4 lens at room temperature, in ProLong® Gold antifade reagent medium from Invitrogen using an internal microscope camera; acquisition software was Zen2008 (Zeiss).

### Protein expression, cell lysis and cell fractionation

Wild type Jak2, Flag-Jak2, K882E mutant, and GFP-Jak2 DNA were expressed in various cells. Cells were washed three times with PBS and lysed with cold lysis buffer (either 1% SDS or 1% Triton X-100 in 1 mM EDTA, 100 mM NaF, 1 mM Na_3_VO_4_, 1 mM PMSF, 10 µg/ml leupeptin and 10 µg/ml aprotinin in PBS). For cell fractionation, cells were washed three times with PBS and then scraped in 1 ml PBS, centrifuged at 10.000 g for 5 min. The pellets were resuspended in 25 mM HEPES, pH 7.9, 5 mM KCl, 0.5 mM MgCl_2_, 1 mM DTT, 1 mM PMSF, 10 µg/ml leupeptin and 10 µg/ml aprotinin. 1 volume of 1% NP-40, 25 mM HEPES pH 7.9, 5 mM KCl, 0.5 mM MgCl_2_, 1 mM DTT, 1 mM PMSF, 10 µg/ml leupeptin and 10 µg/ml aprotinin was added and the lysates were incubated on ice for 15 min. Samples were centrifuged at 500 g for 5 min, and the pellets were washed and centrifuged again at 500 g for 5 min. The pellets were dissolved in 1 volume of 1% SDS lysis buffer.
